# From parental psychological control to hidden emotional pressure: a multimodal review of children's socio-emotional development

**DOI:** 10.3389/fpsyg.2026.1900170

**Published:** 2026-07-06

**Authors:** Lei Jin, Xinyi Jiang, Feng Guo

**Affiliations:** 1 Xi'an Jiaotong-Liverpool University, Suzhou, Jiangsu, China; 2 Mindspring Mental Health Clinic, Shanghai, China

**Keywords:** emotional autonomy, empathy, guilt induction, intrusive parenting, love withdrawal, multimodal affective computing, parental psychological control, social adaptation

## Abstract

Multimodal data processing is becoming increasingly important in neuroscience and perception science because children's emotional and social experiences are rarely expressed through a single observable channel. In parent–child interaction, children may verbally comply with parental expectations while simultaneously showing subtle affective, behavioral, physiological, or neural signs of internal distress. This review examines parental psychological control as a hidden but developmentally significant context for multimodal assessment. Unlike behavioral control, which regulates children's external behavior through rules and supervision, psychological control intrudes into children's inner emotional world through guilt induction, love withdrawal, and intrusive parenting. This review first summarizes how parental psychological control may create hidden emotional pressure and undermine children's socio-emotional development. Guilt induction may increase self-blame and emotional over-responsibility, thereby weakening emotional autonomy. Love withdrawal may make children associate affection with obedience, increasing rejection fear and distorting empathic expression. Intrusive parenting may restrict independent decision-making and social problem-solving, thereby impairing social adaptation. These mechanisms suggest that children exposed to psychological control may appear outwardly calm, obedient, or well-adjusted while internally experiencing emotional inhibition, relational anxiety, and reduced agency. To address this hidden discrepancy, this review highlights the value of multimodal data processing for detecting implicit emotional burden in parent–child interaction. Potential indicators include facial micro-expressions, vocal changes, gaze avoidance, body movement, language patterns, physiological arousal, and neural signals related to emotional reactivity and regulatory effort. By integrating behavioral observation, affective computing, physiological sensing, and neurodevelopmental evidence, multimodal approaches may provide a more sensitive framework for understanding how psychologically controlling parenting shapes children's emotional autonomy, empathy, and social adaptation.

## 1 Introduction

### 1.1 Background

Children's socio-emotional development constitutes a fundamental dimension of psychological growth, involving emotional awareness, emotion regulation, empathy, interpersonal communication, and adaptive participation in family, peer, and school contexts. In this review, the term “children” is used broadly to refer to individuals across childhood and adolescence, unless a specific developmental stage is explicitly discussed, as parental psychological control may influence socio-emotional development throughout these developmental periods. These capacities are not formed in isolation; rather, they emerge through repeated social interactions in which children learn how emotions are expressed, validated, controlled, or suppressed. Within this process, parenting plays a central role by shaping the emotional climate in which children develop self-understanding and relational competence (Lin et al., 2024; Zitzmann et al., 2024; Vasiou et al., 2023). Recent developmental research has therefore increasingly emphasized the need to examine not only overt parenting behaviors, but also the subtle emotional and relational messages embedded in parent–child interaction (Xu et al., 2024; Ricker et al., 2024).

Among different parenting dimensions, parental psychological control has received growing attention as a hidden but developmentally significant form of parenting. Unlike behavioral control, which involves rule-setting, supervision, and guidance of children's external behavior, psychological control intrudes into children's internal psychological world by manipulating emotions, self-evaluation, guilt, attachment needs, and relational security. It is commonly manifested through guilt induction, love withdrawal, shaming, conditional approval, and intrusive interference in children's personal decisions and emotional experiences (Beliveau et al., 2023; Bradshaw et al., 2025; Tang et al., 2024). Although such practices may be justified by parents as concern, protection, discipline, or emotional involvement, they may communicate to children that love, approval, and relational security are conditional upon obedience and emotional compliance (Costa et al., 2025; Wentholt et al., 2025).

A growing body of evidence indicates that parental psychological control is associated with a broad range of maladaptive developmental outcomes, including emotion regulation difficulties, lower self-esteem, anxiety, depressive symptoms, social avoidance, and reduced subjective wellbeing (Beliveau et al., 2023; Deng et al., 2024; Zhu et al., 2024b). Meta-analytic findings suggest that psychologically controlling parenting is reliably linked to poorer emotion regulation in youth, highlighting emotion regulation as a key developmental pathway through which psychological control affects adjustment (Beliveau et al., 2023; Lin et al., 2024). Longitudinal studies further indicate that parental psychological control and adolescent wellbeing may influence each other over time, suggesting that psychological control should be understood as part of a dynamic parent–child developmental process rather than a static parenting trait (Deng et al., 2024; Ye et al., 2026).

### 1.2 Core mechanisms

Although parental psychological control is often treated as a broad parenting construct, its developmental influence may depend on specific controlling practices. One major pathway through which parental psychological control affects children is the disruption of emotional autonomy. Emotional autonomy refers to children's capacity to recognize, express, and regulate their own emotions while maintaining a stable sense of self within close relationships. Guilt induction may directly undermine this capacity by making children feel responsible for parents' disappointment, sacrifice, or distress. When children repeatedly receive messages implying that their choices or emotions hurt their parents, they may learn to suppress their authentic feelings and prioritize parental emotional needs over their own (Ricker et al., 2024; Tang et al., 2024). Over time, this process may increase self-blame, shame, and emotional over-responsibility, while weakening children's ability to distinguish their own emotional states from parental expectations (Zitzmann et al., 2024; Costa et al., 2025).

A second pathway concerns children's empathic expression. Healthy empathy requires sensitivity to others' emotions, but it also depends on self–other differentiation and secure emotional boundaries. Love withdrawal may distort this process by teaching children that affection is conditional and that emotional disagreement may threaten relational security. Children exposed to this form of control may become highly vigilant to parental emotional cues and may appear considerate or compliant; however, their empathic responses may be driven more by fear of rejection than by secure concern for others (Wentholt et al., 2025; Fu et al., 2025). This pattern may later generalize to peer relationships, where children may over-accommodate others, avoid conflict, and struggle to express their own needs (Ye et al., 2026; Chen et al., 2024).

A third pathway involves social adaptation. Intrusive parenting restricts children's opportunities to make decisions, solve interpersonal problems, tolerate manageable frustration, and learn from social feedback. In the short term, intrusive parental involvement may produce outward obedience and behavioral order; however, in the long term, it may reduce children's initiative, social confidence, and problem-solving flexibility (Xu et al., 2024; Hu et al., 2025). Recent work based on self-determination theory suggests that autonomy-supportive parenting and psychologically controlling parenting represent distinct developmental processes: autonomy support is positively associated with children's wellbeing, whereas psychological control is more strongly linked to ill-being and maladjustment (Bradshaw et al., 2025; Chen et al., 2024). This distinction indicates that reducing psychological control is not sufficient; children also need relational environments that validate their perspectives and support independent social exploration.

### 1.3 Research gap

Despite these advances, several limitations remain in the existing literature. First, many studies continue to rely heavily on questionnaires and retrospective reports, which may not fully capture the implicit emotional processes occurring during real-time parent–child interaction. Psychological control is often subtle, relational, and difficult to observe directly. Children may verbally report family harmony or appear behaviorally compliant while internally experiencing fear, guilt, shame, emotional inhibition, or reduced agency. Therefore, conventional self-report and behavioral measures may underestimate the hidden emotional burden caused by psychologically controlling parenting (Wentholt et al., 2025; Selman and Dilworth-Bart, 2024).

Second, psychological control is often treated as a global construct, while the potentially different effects of guilt induction, love withdrawal, and intrusive parenting remain insufficiently differentiated. This is problematic because these practices may operate through different socio-emotional mechanisms. Guilt induction may primarily weaken emotional autonomy by increasing self-blame and over-responsibility; love withdrawal may distort empathic expression by creating fear of rejection; and intrusive parenting may undermine social adaptation by restricting children's independent decision-making and social problem-solving opportunities (Beliveau et al., 2023; Fu et al., 2025; Wentholt et al., 2025). A more fine-grained review is therefore needed to clarify how each form of psychological control contributes to specific developmental outcomes.

Third, the existing literature has not sufficiently connected developmental psychology with multimodal affective and neurodevelopmental methods. During psychologically controlling interactions, children may verbally comply while simultaneously showing subtle facial tension, reduced vocal energy, gaze avoidance, delayed responses, physiological arousal, or neural markers of stress and regulatory effort. Integrating facial expression, speech, language, body movement, physiological signals, and neural indicators may therefore help reveal children's implicit emotional burden more sensitively than single-method assessment (**?**Zhu et al., 2024). This approach is particularly important for younger children, who may lack the verbal ability or relational safety to explicitly report distress (Vasiou et al., 2023; Vatou et al., 2026).

### 1.4 Aims and contributions of this review

In this review, hidden emotional pressure refers to an internal emotional burden that remains largely unexpressed or unobservable despite outward compliance. Unlike emotional distress, emotional suppression, psychological stress, or internalizing symptoms, hidden emotional pressure emphasizes the underlying and often concealed emotional burden that may contribute to these experiences and outcomes over time. Accordingly, this review aims to synthesize recent evidence on the hidden impact of parental psychological control on children's socio-emotional development Specifically, it focuses on three representative forms of psychological control: guilt induction, love withdrawal, and intrusive parenting. It further examines how these practices influence three core developmental outcomes: emotional autonomy, empathic expression, and social adaptation. By integrating recent findings from parenting research, child development, emotion regulation, family studies, and multimodal affective science, this review seeks to clarify the mechanisms through which parental psychological control affects children's long-term socio-emotional competence and to propose future directions for more sensitive assessment and intervention (Beliveau et al., 2023; Bradshaw et al., 2025; Lin et al., 2024; Xu et al., 2024). These three forms were selected because they are among the most widely studied and conceptually distinct manifestations of parental psychological control in the developmental literature. Other related constructs, such as shaming, conditional regard, invalidation, and parental criticism, are also relevant but often overlap with these broader mechanisms and therefore fall outside the primary scope of the present review.

This article is intended as a narrative and conceptual review. Rather than providing an exhaustive systematic synthesis of the literature, the review aims to integrate evidence from developmental psychology, parenting research, and multimodal affective science to develop a conceptual framework for understanding how parental psychological control may contribute to hidden emotional pressure and socio-emotional development. This approach is appropriate because the review seeks to synthesize mechanisms, theoretical perspectives, and future research directions across multiple disciplines. The present review makes three main contributions. First, it shifts the focus from general parenting styles to parental psychological control as a hidden form of emotional and relational intrusion. Rather than treating psychological control as merely one dimension of parenting, this review emphasizes its unique developmental significance in shaping children's internal emotional world, self-boundaries, and relational expectations. Second, it distinguishes guilt induction, love withdrawal, and intrusive parenting as three representative mechanisms through which psychological control may influence children. This distinction allows a more precise understanding of how different parental practices may undermine emotional autonomy, empathic expression, and social adaptation. These outcomes were prioritized because they represent complementary dimensions of socio-emotional development that are directly relevant to psychologically controlling parenting. Emotional autonomy reflects children's capacity to develop an independent emotional self, empathic expression reflects interpersonal and relational functioning, and social adaptation reflects broader functioning within social environments. Related constructs such as emotion regulation, internalizing symptoms, peer competence, and wellbeing remain important but are considered associated mechanisms or broader developmental outcomes rather than the primary focus of the present review. Third, it introduces a multimodal and neurodevelopmental perspective for future research, suggesting that facial expression, vocal features, behavioral patterns, physiological signals, and neural indicators may help detect hidden emotional pressure that is not easily captured by questionnaires alone.

### 1.5 Organization of the review

**Figure 1:**
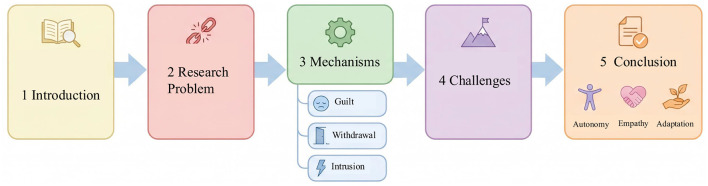
Overall structure of the review on parental psychological control and children's socio-emotional development. The review first introduces the research background, then identifies the research problem, analyzes the mechanisms of guilt induction, love withdrawal, and intrusive parenting, discusses current challenges, and finally summarizes implications for emotional autonomy, empathy, and social adaptation.

The remainder of this review is organized as follows. Section 2 defines the research problem and evaluates the limitations of existing reviews, with particular attention to the need for a more mechanism-oriented and socio-emotional perspective. Section 3 examines the mechanisms linking parental psychological control to children's socio-emotional development, focusing on guilt induction, love withdrawal, intrusive parenting, and their effects on emotional autonomy, empathic expression, and social adaptation. Section 4 discusses current challenges and potential solutions, including conceptual ambiguity, measurement limitations, causal uncertainty, translational barriers, and the promise of multimodal assessment. Finally, Section 5 concludes the review by summarizing the hidden developmental consequences of parental psychological control and highlighting future directions for research, assessment, and intervention. The overall structure of the review is illustrated in Figure 1.

### 1.6 Literature identification strategy

The literature included in this narrative review was identified through searches of major academic databases, including Web of Science, Scopus, PubMed, and Google Scholar. Representative search terms included combinations of “parental psychological control,” “guilt induction,” “love withdrawal,” “intrusive parenting,” “emotion regulation,” “socio-emotional development,” “multimodal assessment,” and related keywords. Priority was given to peer-reviewed articles published in recent years, while seminal theoretical and empirical studies were also included when relevant. Studies focusing on parental psychological control, children's or adolescents' socio-emotional development, and multimodal approaches to emotional assessment were considered. Publications not directly related to the review themes or lacking sufficient relevance to the proposed conceptual framework were excluded.

## 2 Research problem and limitations of existing reviews

### 2.1 Defining the research problem: from parental control to hidden socio-emotional consequences

Parental psychological control has become an increasingly important topic in developmental psychology, family studies, and child mental health research. Although parents may use psychological control in the name of discipline, protection, or emotional involvement, this form of parenting intrudes into children's internal psychological space and may weaken their autonomy, self-worth, and emotional security. Recent empirical studies and meta-analyses have shown that parental psychological control is associated with emotion regulation difficulties, lower self-esteem, anxiety, depression, social avoidance, and reduced wellbeing among children and adolescents (Beliveau et al., 2023; Lin et al., 2024; Tang et al., 2024; Deng et al., 2024). However, the developmental mechanisms through which psychological control affects children's socio-emotional competence remain insufficiently integrated.

The central research problem of this review is therefore not merely whether parental psychological control is harmful, but how its specific forms produce hidden socio-emotional consequences. In particular, guilt induction, love withdrawal, and intrusive parenting may affect children through partially different pathways. Guilt induction may make children feel excessively responsible for parental distress; love withdrawal may lead children to associate affection with obedience; and intrusive parenting may reduce children's opportunities for independent decision-making and social problem solving (Bradshaw et al., 2025; Ricker et al., 2024; Costa et al., 2025). These mechanisms suggest that psychological control should not be treated as a single undifferentiated construct, but as a set of emotionally manipulative practices with distinct developmental implications.

This issue is especially important because the effects of psychological control are often hidden beneath outward compliance. Children exposed to psychologically controlling parenting may appear obedient, mature, considerate, or academically well-adjusted, while internally experiencing self-blame, fear of rejection, emotional suppression, and reduced agency. Therefore, the key problem is not only behavioral maladjustment, but also the erosion of children's emotional autonomy, authentic empathic expression, and adaptive social functioning. This review thus focuses on the implicit socio-emotional costs of psychological control rather than limiting the discussion to overt symptoms or behavioral problems.

### 2.2 Existing reviews: contributions to parenting, emotion regulation, and child adjustment

Existing reviews have provided a strong foundation for understanding the association between parenting and child adjustment. For example, recent meta-analytic work has demonstrated that child emotion regulation mediates the association between parenting-related family factors and psychopathology (Zhao et al., 2026; Sun et al., 2026; Wang et al., 2025a). Another meta-analysis specifically showed that parental psychological control is reliably related to poorer youth emotion regulation, indicating that emotion regulation is a key pathway through which controlling parenting may influence child development (Beliveau et al., 2023; Lin et al., 2026; Zhu et al., 2025). In addition, broader reviews on parenting, emotion regulation, and child socio-emotional development have highlighted the importance of family emotional climate, parental mental health, and parenting styles in shaping children's adjustment (Zitzmann et al., 2024; Honda et al., 2023; Vasiou et al., 2023).

These studies have made several important contributions. First, they confirm that parenting is not only a behavioral management process, but also an emotional and relational context for child development. Second, they show that emotion regulation plays a central role in linking parenting practices to mental health outcomes. Third, they suggest that psychological control is different from autonomy support and behavioral control, because it directly targets children's internal experience rather than only guiding external behavior (Bradshaw et al., 2025; Xu et al., 2024; Wang et al., 2025b). Together, these findings provide a strong empirical foundation for examining psychological control as a distinct developmental risk factor.

Nevertheless, most existing reviews have focused on general parenting dimensions, broad emotional adjustment, or psychopathological outcomes. They have been highly valuable for establishing overall associations, but they do not fully explain how specific forms of psychological control shape different aspects of children's socio-emotional competence. For example, guilt induction, love withdrawal, and intrusive parenting are often grouped together under the broad label of psychological control, even though they may involve different emotional messages, relational threats, and developmental consequences (Tang et al., 2024; Wentholt et al., 2025; Fu et al., 2025). This limits the field's ability to identify precise mechanisms and design targeted interventions.

Table 1 summarizes representative recent reviews and empirical syntheses relevant to the present topic. Overall, these studies provide important evidence that parenting is closely related to children's emotion regulation, mental health, and socio-emotional development. However, they also reveal a clear gap: few reviews have systematically examined how specific forms of parental psychological control influence children's emotional autonomy, empathic expression, and social adaptation from a multimodal and developmental perspective.

**Table 1:** Representative recent reviews and empirical syntheses related to parental psychological control and children's socio-emotional development.

References	Type	Main focus	Key contribution	Remaining limitation
Beliveau et al. (2023)	Meta-analysis	Parental psychological control and youth emotion regulation	Demonstrated a reliable association between psychological control and poorer emotion regulation in youth.	Focused mainly on emotion regulation, with less attention to empathy, emotional autonomy, and social adaptation.
Lin et al. (2024)	Meta-analysis	Parenting, emotion regulation, and child psychopathology	Identified child emotion regulation as a key mediator between parenting-related family factors and psychopathology.	Examined broader family factors, but did not deeply differentiate specific forms of psychological control.
Bradshaw et al. (2025)	Meta-analysis	Autonomy support and psychological control across cultures	Clarified the dual-process model of autonomy-supportive and psychologically controlling parenting.	Focused mainly on wellbeing and ill-being, with limited discussion of hidden socio-emotional mechanisms.
Zitzmann et al. (2024)	Systematic review	Parenting, emotion regulation, and psychopathology	Integrated evidence linking parenting practices with children's emotion regulation difficulties and psychopathological outcomes.	Provided limited discussion of guilt induction, love withdrawal, and intrusive parenting as distinct mechanisms.
Honda et al. (2023)	Systematic review and meta-analysis	Parental mental health and children's socio-emotional development	Highlighted parental mental health as an important environmental factor shaping children's socio-emotional outcomes.	Focused on parental mental health rather than psychological control as a specific parenting practice.
Vasiou et al. (2023)	Empirical synthesis	Parenting style patterns and children's socio-emotional skills	Emphasized multidimensional patterns of parenting styles in children's socio-emotional development.	Did not specifically examine hidden emotional manipulation within parent–child interaction.

### 2.3 Unresolved gaps and the contribution of the present review

Despite these advances, several limitations remain in the existing review literature. First, many reviews focus on general parenting dimensions, such as warmth, harshness, autonomy support, or behavioral control, while psychological control is often discussed as only one component within a broader parenting framework (Bradshaw et al., 2025; Vasiou et al., 2023; Zhang et al., 2025). As a result, the unique features of psychological control—especially its hidden, relational, and emotionally manipulative nature—are not always sufficiently emphasized. This is problematic because psychological control can be difficult to identify in daily life and may be mistakenly interpreted as care, concern, or parental responsibility.

Second, existing reviews often emphasize psychopathological outcomes, such as depression, anxiety, and externalizing problems, but pay less attention to socio-emotional competence as a developmental outcome in its own right. Although mental health symptoms are important, children exposed to psychological control may also experience more subtle difficulties, including reduced emotional autonomy, distorted empathy, poor boundary awareness, and social over-compliance (Tang et al., 2024; Li et al., 2024; Zhu et al., 2024b). These outcomes may not always appear as clinical symptoms, but they can affect children's long-term interpersonal development and adaptive functioning.

Third, current reviews tend to rely heavily on questionnaire-based evidence and self-report measures. While these methods are valuable for large-scale research, they may not fully capture the implicit emotional processes that occur during real-time parent–child interactions (Wentholt et al., 2025). Children under psychological control may verbally comply with parents while simultaneously showing subtle signs of distress, such as facial tension, reduced vocal expressiveness, gaze avoidance, physiological arousal, or delayed responses. Therefore, traditional self-report and behavioral questionnaires may underestimate the hidden emotional burden associated with psychological control.

Fourth, existing studies and reviews have not fully distinguished among guilt induction, love withdrawal, and intrusive parenting. These practices are often grouped under the broad label of psychological control, but they may operate through different developmental mechanisms. Guilt induction may primarily target children's emotional responsibility and self-blame; love withdrawal may threaten relational security and attachment; and intrusive parenting may constrain autonomy development and social competence (Beliveau et al., 2023; Fu et al., 2025; Wentholt et al., 2025; Tian et al., 2026). A more fine-grained review is needed to clarify how each form of psychological control contributes to specific socio-emotional outcomes.

Based on these gaps, the present review aims to make three contributions. First, it shifts the focus from general parenting styles to parental psychological control as a hidden form of emotional and relational intrusion. Second, it distinguishes guilt induction, love withdrawal, and intrusive parenting as three representative mechanisms through which psychological control may affect children. Third, it links these parenting practices to three core dimensions of socio-emotional competence: emotional autonomy, empathic expression, and social adaptation. By integrating recent evidence from developmental psychology, child mental health, family studies, and multimodal affective science, this review provides a more mechanism-oriented framework for understanding the hidden impact of parental psychological control on children's socio-emotional development (Xu et al., 2024; Costa et al., 2025; Fu et al., 2025).

## 3 Mechanisms linking parental psychological control to children's socio-emotional development

This section synthesizes the developmental pathways linking parental psychological control to children's socio-emotional development. To facilitate the integration of findings across different mechanisms, several conceptual equations are introduced throughout this section. These equations are intended solely as illustrative representations of the relationships discussed in the reviewed literature and should not be interpreted as statistically estimated or empirically validated models.

### 3.1 Conceptualizing parental psychological control as intrusion into the child's inner world

Parental psychological control refers to parenting practices that intrude into children's psychological and emotional world by manipulating their thoughts, feelings, self-evaluation, and attachment needs. Unlike behavioral control, which may provide structure, supervision, and safety boundaries, psychological control targets children's internal experience and often operates through emotional pressure, conditional approval, guilt, shame, and relational threat (Beliveau et al., 2023; Bradshaw et al., 2025; Xu et al., 2024). This distinction is essential because behavioral control may support adaptive functioning when it is warm, consistent, and developmentally appropriate, whereas psychological control tends to undermine children's autonomy and emotional security even when it is framed as care or protection (Ricker et al., 2024; Tang et al., 2024; Zhu et al., 2026b). For example, behavioral control may involve setting household rules, monitoring children's activities, or supervising age-appropriate responsibilities. In contrast, psychological control may involve making children feel guilty for disappointing their parents, withdrawing affection when expectations are not met, providing approval only when children comply, or interfering with children's thoughts, emotions, and personal decisions.

To clarify the construct, this review conceptualizes parental psychological control as a multidimensional form of emotional and relational intrusion. Rather than treating it as a single global parenting style, it is more useful to distinguish its major forms and their developmental meanings. As summarized in Table 2, guilt induction, love withdrawal, and intrusive parenting share the common feature of restricting children's psychological autonomy, but they communicate different emotional messages and may create different developmental risks.

**Table 2:** Core forms of parental psychological control and their developmental implications.

Form	Typical parental behaviors	Core psychological message	Potential developmental risk	Representative evidence
Guilt induction	Emphasizing parental sacrifice, disappointment, suffering, or emotional burden to influence children's behavior.	“My emotions are your responsibility.”	Self-blame, shame, emotional over-responsibility, and weakened emotional autonomy.	Beliveau et al., 2023; Tang et al., 2024; Ricker et al., 2024
Love withdrawal	Reducing warmth, attention, affection, or emotional availability when children fail to meet expectations.	“Affection depends on obedience.”	Relational anxiety, insecure attachment expectations, anxious empathy, and fear of rejection.	Costa et al., 2025; Wentholt et al., 2025; Fu et al., 2025
Intrusive parenting	Excessively interfering with children's choices, peer relations, emotional reactions, and problem-solving processes.	“Your own judgment is not reliable.”	Reduced agency, poor social confidence, dependence on external approval, and weaker social adaptation.	Xu et al., 2024; Bradshaw et al., 2025; Ye et al., 2026

Recent research has shown that parental psychological control is associated with a wide range of developmental risks, including emotion regulation difficulties, depressive symptoms, anxiety, lower self-esteem, reduced subjective wellbeing, and social maladjustment (Lin et al., 2024; Deng et al., 2024; Zhu et al., 2024b). However, psychological control should not be understood only as a predictor of psychopathology. More importantly, it may gradually reshape children's socio-emotional development by changing how they understand their own emotions, how they respond to others' emotions, and how they participate in social relationships (Zitzmann et al., 2024; Vasiou et al., 2023). Therefore, this review treats psychological control as a developmental process affecting three core domains: emotional autonomy, empathic expression, and social adaptation.

At the conceptual level, the developmental influence of parental psychological control can be expressed as follows the conceptual relationships discussed in this section are summarized in Equations 1–6:


$$\begin{array}{l} SEC_{i}=f\left(EA_{i},EE_{i},SA_{i}\right), \end{array}$$ (1)

where *SEC*_*i*_ denotes the socio-emotional competence of child *i*, *EA*_*i*_ refers to emotional autonomy, *EE*_*i*_ refers to empathic expression, and *SA*_*i*_ refers to social adaptation. In this review, parental psychological control is assumed to affect socio-emotional competence by disrupting these three interrelated domains.

More specifically, the hidden influence of psychological control can be represented as a mediated pathway:


$$\begin{array}{l} PPC\to HEP\to SEC, \end{array}$$ (2)

where *PPC* denotes parental psychological control, *HEP* denotes hidden emotional pressure, and *SEC* denotes children's socio-emotional competence. This formulation highlights that the developmental harm of psychological control is not always directly visible. Children may appear obedient or well-adjusted while internally experiencing self-blame, fear of rejection, emotional inhibition, or reduced agency (Wentholt et al., 2025; Costa et al., 2025).

### 3.2 Guilt induction and the weakening of emotional autonomy

Guilt induction is one of the most representative forms of parental psychological control. It occurs when parents emphasize their own sacrifice, disappointment, suffering, or emotional burden in order to influence children's behavior. Rather than helping children understand the consequences of their actions, guilt induction often makes children feel responsible for parents' negative emotions (Beliveau et al., 2023; Tang et al., 2024; Zhu et al., 2026a). For example, when parents repeatedly communicate that a child's disagreement, failure, or independent choice causes parental sadness or disappointment, the child may gradually internalize the belief that maintaining parental emotional stability is their personal responsibility.

This mechanism directly threatens children's emotional autonomy. Emotional autonomy requires children to recognize, interpret, and express their own feelings while maintaining appropriate emotional boundaries within close relationships. Under guilt induction, however, children may learn to prioritize parents' emotional needs over their own. They may suppress anger, sadness, or disagreement because these emotions are perceived as harmful to the parent–child relationship (Ricker et al., 2024; Lin et al., 2024). Over time, this process may weaken children's ability to distinguish between their own emotional states and the emotions imposed by parental expectations.

The effect of guilt induction can be described as an increase in emotional responsibility imposed on the child:


$$\begin{array}{l} HEP_{guilt}=\alpha_{1}ER+\alpha_{2}SB+\alpha_{3}ES, \end{array}$$ (3)

where *HEP*_*guilt*_ denotes hidden emotional pressure produced by guilt induction, *ER* denotes excessive emotional responsibility, *SB* denotes self-blame, and *ES* denotes emotional suppression. This formula is not intended as a statistical model, but as a conceptual expression of how guilt induction may convert parental distress into children's internalized emotional burden.

Guilt induction may also increase shame and self-criticism. Children who are repeatedly made to feel responsible for parental distress may interpret ordinary developmental needs, such as autonomy, privacy, or peer affiliation, as selfish or morally wrong. This pattern is especially harmful during middle childhood and adolescence, when children begin to develop stronger self-evaluation, identity, and independent emotional judgment (Deng et al., 2024; Luo et al., 2026). Instead of developing a stable sense of emotional agency, children may become overly concerned with whether their emotions are acceptable to parents.

### 3.3 Love withdrawal and the distortion of empathic expression

Love withdrawal refers to parental behaviors in which affection, warmth, attention, or emotional availability is reduced when children fail to meet parental expectations. It may appear as silence, coldness, emotional distance, disappointment, or refusal to provide comfort. Although it may not involve direct verbal punishment, love withdrawal communicates a powerful relational message: parental affection is conditional upon obedience, achievement, or emotional conformity (Costa et al., 2025; Wentholt et al., 2025). This message can shape children's understanding of close relationships and influence how they express empathy toward others.

Healthy empathy requires both sensitivity to others' emotions and clear self–other differentiation. Children need to understand others' feelings without losing their own emotional boundaries. Love withdrawal may distort this process by making children hypervigilant to relational cues. Children may become highly skilled at detecting parental dissatisfaction, anger, or emotional distance, but this sensitivity may be driven by anxiety rather than secure concern (Fu et al., 2025; Vasiou et al., 2023). As a result, they may develop a form of anxious empathy, in which caring for others is closely tied to fear of rejection.

The developmental pathway of love withdrawal can be summarized as:


$$\begin{array}{l} HEP_{withdrawal}=\beta_{1}RF+\beta_{2}RA-\beta_{3}RS, \end{array}$$ (4)

where *RF* denotes rejection fear, *RA* denotes relational anxiety, and *RS* denotes relational security. This formulation suggests that love withdrawal increases hidden emotional pressure by increasing fear of rejection and relational anxiety while weakening the child's sense of secure acceptance.

This pattern may later generalize to peer relationships. Children who have learned that affection can be withdrawn may become overly compliant, conflict-avoidant, or excessively responsible for others' feelings. They may appear considerate and emotionally mature, but their empathic behavior may lack autonomy and reciprocity (Chen et al., 2024; Ye et al., 2026). Thus, love withdrawal may not reduce empathic sensitivity; rather, it may transform empathy into a defensive relational strategy.

### 3.4 Intrusive parenting and the erosion of social adaptation

Intrusive parenting involves excessive parental interference in children's decisions, preferences, peer relationships, emotional reactions, and problem-solving processes. Unlike supportive involvement, which provides guidance while respecting the child's perspective, intrusive parenting limits children's opportunities to make choices, experience manageable failure, negotiate conflicts, and learn from social feedback (Xu et al., 2024; Bradshaw et al., 2025). It may include over-directing children's activities, invalidating their preferences, speaking on their behalf, controlling friendships, or imposing parental goals as the child's own.

Social adaptation requires children to develop initiative, confidence, flexible problem solving, and the ability to coordinate personal needs with social expectations. Intrusive parenting may undermine these capacities by reducing children's sense of agency. When parents repeatedly take over decisions or correct children's social responses, children may become dependent on external approval and less confident in their own judgment (Hu et al., 2025; Song et al., 2025). This may lead to passivity, avoidance, or excessive reliance on adults in peer and school contexts.

The social-developmental cost of intrusive parenting may be represented as:


$$\begin{array}{l} SA=\gamma_{1}AG+\gamma_{2}SP+\gamma_{3}SC-\gamma_{4}PI, \end{array}$$ (5)

where *SA* denotes social adaptation, *AG* denotes agency, *SP* denotes social problem-solving, *SC* denotes social confidence, and *PI* denotes parental intrusion. This conceptual expression indicates that intrusive parenting may reduce social adaptation by limiting the child's agency, practice opportunities, and confidence in independent social judgment.

The effect of intrusive parenting is often paradoxical. In the short term, high parental interference may produce order, compliance, and fewer visible mistakes. However, long-term adaptation requires children to practice independent decision-making and social problem solving. Children who have limited autonomy may struggle when they encounter ambiguous social situations, peer rejection, competition, or interpersonal conflict (Ye et al., 2026; Bradshaw et al., 2025). In this sense, intrusive parenting may create a surface appearance of competence while weakening the internal resources needed for flexible social functioning.

After examining the three pathways separately, Table 3 integrates them into a single mechanism-oriented framework. This table shows that different psychological control practices may target different psychological processes, but all of them may ultimately contribute to hidden socio-emotional vulnerability.

**Table 3:** Mechanistic pathways linking parental psychological control to children's socio-emotional outcomes.

Practice	Immediate emotional process	Affected domain	Possible long-term consequence	Supporting studies
Guilt induction	Children internalize parental distress and become overly responsible for maintaining parental emotional stability.	Emotional autonomy.	Self-blame, emotional suppression, weak emotional boundaries, and internalizing vulnerability.	Beliveau et al., 2023; Ricker et al., 2024; Deng et al., 2024
Love withdrawal	Children perceive affection as conditional and become hypervigilant to signs of rejection or relational distance.	Empathic expression.	Anxious empathy, conflict avoidance, over-compliance, and difficulty expressing personal needs.	Costa et al., 2025; Wentholt et al., 2025; Fu et al., 2025
Intrusive parenting	Children receive fewer opportunities to make decisions, solve problems, and learn from social feedback.	Social adaptation.	Reduced agency, dependence on external approval, poor social confidence, and difficulty handling peer conflicts.	Xu et al., 2024; Bradshaw et al., 2025; Ye et al., 2026

### 3.5 An integrated developmental pathway: from hidden emotional pressure to socio-emotional vulnerability

Taken together, guilt induction, love withdrawal, and intrusive parenting may influence children's socio-emotional development through partially distinct but interconnected pathways. Guilt induction primarily weakens emotional autonomy by increasing self-blame and emotional over-responsibility. Love withdrawal distorts empathic expression by linking care and compliance with fear of rejection. Intrusive parenting erodes social adaptation by limiting children's opportunities for independent decision-making and interpersonal problem solving (Beliveau et al., 2023; Costa et al., 2025; Wentholt et al., 2025). These mechanisms may operate simultaneously within the same family environment and may reinforce each other over time.

The integrated pathway can be summarized as:


$$\begin{array}{l} SEC=\delta_{1}EA+\delta_{2}EE+\delta_{3}SA-\delta_{4}HEP, \end{array}$$ (6)

where *SEC* denotes socio-emotional competence, *EA* denotes emotional autonomy, *EE* denotes empathic expression, *SA* denotes social adaptation, and *HEP* denotes hidden emotional pressure. This expression highlights that children's socio-emotional competence depends not only on positive developmental capacities but also on the reduction of hidden emotional pressure produced by psychologically controlling parenting.

One important feature of this pathway is its hidden nature. Children exposed to psychological control may regulate themselves in ways that are socially approved but psychologically costly. They may become quiet, obedient, helpful, and highly responsive to others' emotions, yet internally experience anxiety, shame, low autonomy, and difficulty asserting their own needs (Tang et al., 2024; Deng et al., 2024; Zhu et al., 2024b). Therefore, children's surface-level compliance should not be treated as sufficient evidence of healthy socio-emotional development.

This hidden process suggests that traditional questionnaires and behavioral observations may not be sufficient. Children may verbally agree with parents while showing subtle facial tension, gaze avoidance, reduced vocal energy, delayed responses, increased physiological arousal, or neural markers of regulatory effort (Zitzmann et al., 2024). Table 4 therefore summarizes potential multimodal indicators that may help future studies capture the discrepancy between outward compliance and internal distress. Although these multimodal indicators are not uniquely associated with any single parenting practice, certain patterns may be particularly relevant to specific forms of psychological control. For example, guilt induction may be reflected in linguistic indicators such as self-blame, excessive apology, or reduced self-referential agency. Love withdrawal may be associated with facial and behavioral signs of relational anxiety, including gaze avoidance, reduced positive affect, and heightened vigilance to interpersonal cues. Intrusive parenting may be reflected in reduced behavioral initiative, hesitant responses, and physiological indicators of heightened regulatory effort during decision-making or disagreement situations.

**Table 4:** Potential multimodal indicators of hidden emotional pressure during psychologically controlling parent–child interactions.

Modality	Possible signal	Interpretive value	Relevance to psychological control	Related evidence
Facial expression	Facial tension, reduced positive affect, forced smiling, and micro-expressions of sadness or fear.	Reveals affective discomfort that is not verbally expressed.	Useful for detecting distress beneath outward compliance.	Zitzmann et al., 2024
Speech and voice	Lower vocal energy, longer pauses, hesitant responses, reduced pitch variability, and shorter utterances.	Indicates emotional inhibition, uncertainty, or fear of disagreement.	Relevant to guilt induction and love withdrawal contexts.	Gilgoff et al., 2024
Gaze and body movement	Gaze avoidance, bodily withdrawal, reduced gesture, rigid posture, and delayed response orientation.	Reflects avoidance, submission, or relational anxiety.	Useful for identifying defensive adaptation during parent–child conflict.	Wentholt et al., 2025; Costa et al., 2025
Physiological signals	Increased heart rate, reduced heart rate variability, electrodermal activity, and stress-related arousal.	Captures internal stress even when behavior appears calm.	Helps distinguish true adjustment from suppressed distress.	Gilgoff et al., 2024; Zitzmann et al., 2024
Language content	Frequent apology, self-blame, excessive agreement, uncertainty markers, and reduced self-referential agency.	Reveals internalized guilt, self-silencing, or dependence on parental approval.	Directly relevant to emotional autonomy and boundary formation.	Beliveau et al., 2023; Li et al., 2024

Another important feature is developmental accumulation. The consequences of psychological control may not appear immediately but may accumulate across repeated parent–child interactions. In early childhood, psychological control may disrupt emotion understanding and attachment security. In middle childhood, it may affect self-evaluation, peer participation, and school adaptation. In adolescence, it may intensify conflicts around autonomy, identity, and relational boundaries (Lin et al., 2024; Honda et al., 2023; McAloon and Armstrong, 2024). This developmental perspective helps explain why psychological control can be associated with both internalizing symptoms and broader socio-emotional difficulties.

Accordingly, this review proposes that parental psychological control affects children's socio-emotional development through a cascading pathway: psychologically controlling parenting practices create hidden emotional pressure; this pressure disrupts emotional autonomy, empathic expression, and social adaptation; and these socio-emotional vulnerabilities may increase long-term risk for internalizing problems, relational insecurity, and maladaptive coping (Luo et al., 2026; Selman and Dilworth-Bart, 2024). This pathway highlights the need for future research to move beyond global measures of parenting and to examine the specific emotional mechanisms through which psychological control shapes children's developmental outcomes.

**Figure 2:**
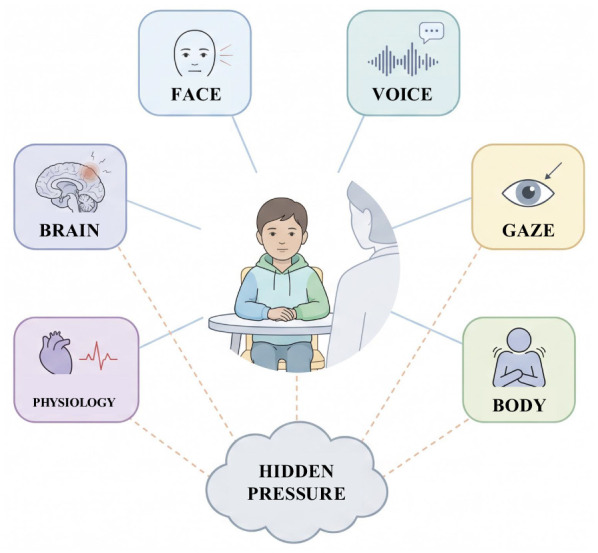
Multimodal indicators of hidden emotional pressure during psychologically controlling parent–child interactions. Facial expression, voice, gaze, body movement, physiological responses, and neural signals may provide complementary evidence for detecting children's internal emotional burden beneath outward compliance.

This hidden process suggests that traditional questionnaires and behavioral observations may not be sufficient. Children may verbally agree with parents while showing subtle facial tension, gaze avoidance, reduced vocal energy, delayed responses, increased physiological arousal, or neural markers of regulatory effort (Zitzmann et al., 2024). As illustrated in Figure 2, multimodal indicators such as facial expression, voice, gaze, body movement, physiological responses, and neural signals may help reveal hidden emotional pressure that is not directly observable through verbal reports alone.

Table 4 further summarizes potential multimodal indicators that may help future studies capture the discrepancy between outward compliance and internal distress.

## 4 Current challenges and potential solutions

Although recent studies have substantially advanced our understanding of parental psychological control and children's socio-emotional development, several unresolved challenges remain. These challenges are not merely methodological; they also concern how psychological control is conceptualized, how developmental mechanisms are identified, and how research findings can be translated into assessment and intervention. In particular, three issues require further attention: the measurement of hidden psychological control, the identification of causal and developmental mechanisms, and the integration of multimodal evidence into family-based prevention and intervention.

### 4.1 Challenge 1: conceptual ambiguity and measurement limitations

A primary challenge concerns the conceptual and measurement boundaries of parental psychological control. Psychological control is often defined as parental intrusion into children's thoughts, emotions, self-evaluation, and relational security. However, in empirical studies, it is sometimes measured as a broad and global parenting dimension, without adequately distinguishing guilt induction, love withdrawal, shaming, conditional approval, and intrusive parenting. This creates conceptual ambiguity because different forms of psychological control may affect children through different socio-emotional pathways (Tang et al., 2024; Deng et al., 2024; Ricker et al., 2024). For example, guilt induction may primarily increase children's self-blame and emotional over-responsibility, whereas love withdrawal may threaten relational security and intrusive parenting may restrict autonomy development.

Another measurement problem is the heavy reliance on self-report questionnaires. Although questionnaires are efficient and useful for large samples, they are vulnerable to recall bias, social desirability, and limited introspective accuracy. Children may normalize psychologically controlling parenting, especially when such practices are culturally framed as care, sacrifice, or discipline. Parents may also underreport controlling practices because they perceive them as responsible involvement rather than emotional intrusion (Xu et al., 2024; Bradshaw et al., 2025). As a result, the hidden emotional burden of psychological control may be underestimated.

A further difficulty lies in distinguishing psychological control from related parenting constructs, such as behavioral control, parental involvement, strictness, and autonomy support. Behavioral control may be adaptive when it provides clear rules and age-appropriate structure, whereas psychological control undermines children's inner autonomy by manipulating emotions and attachment needs. However, these dimensions may overlap in everyday parenting, especially in cultural contexts where obedience, academic achievement, and family obligation are strongly emphasized (Bradshaw et al., 2025; Xu et al., 2024). Without precise measurement, researchers may incorrectly interpret parental guidance as psychological control or overlook controlling behaviors embedded in apparently supportive parenting.

Several solutions are needed. First, future studies should use multidimensional instruments that separately assess guilt induction, love withdrawal, intrusive interference, shaming, and conditional regard. Second, multi-informant designs should be encouraged, combining reports from children, parents, teachers, and independent observers. Third, observational coding of parent–child interaction should be used to capture real-time emotional pressure, especially in conflict, decision-making, and achievement-related contexts (Wentholt et al., 2025). Finally, studies should report measurement invariance across age, gender, and cultural groups to ensure that psychological control is interpreted consistently across developmental and sociocultural contexts.

### 4.2 Challenge 2: unclear mechanisms and causal pathways

A second challenge concerns the identification of mechanisms and causal pathways. Although a growing body of evidence links parental psychological control to emotion regulation difficulties, lower self-esteem, anxiety, depression, social avoidance, and reduced wellbeing, much of the literature remains correlational. This makes it difficult to determine whether psychological control leads to children's socio-emotional difficulties, whether children's difficulties elicit more controlling parenting, or whether both are shaped by broader family stressors (Lin et al., 2024; Deng et al., 2024; Zhu et al., 2024b). Recent longitudinal evidence suggests that parental psychological control and adolescent adjustment may influence each other over time, indicating a reciprocal developmental process rather than a simple one-way effect (Deng et al., 2024; Luo et al., 2026).

The mechanism problem is also related to the diversity of child outcomes. Existing research often focuses on psychopathological symptoms, such as depression and anxiety, while giving less attention to more subtle socio-emotional outcomes, including emotional autonomy, empathic expression, boundary awareness, interpersonal confidence, and social problem-solving. However, psychological control may first appear as internal emotional strain rather than overt clinical symptoms. Children may become compliant, quiet, and sensitive to others' emotions, yet simultaneously experience self-silencing, shame, fear of rejection, and reduced agency (Fu et al., 2025; Li et al., 2024; Ye et al., 2026). Therefore, focusing only on clinical symptoms may miss important developmental changes.

Another unresolved issue is heterogeneity. The effect of psychological control may vary by developmental stage, child temperament, parent mental health, family stress, culture, and gender. For instance, young children may experience love withdrawal as a direct threat to attachment security, whereas adolescents may respond to the same practice with psychological reactance or social withdrawal. Similarly, children with high emotional sensitivity may be more vulnerable to guilt induction, while children with stronger self-regulation may show greater resilience (Lin et al., 2024; Luo et al., 2026). These differences indicate that the same parenting behavior may produce different outcomes depending on individual and contextual conditions.

To address these problems, future research should adopt stronger longitudinal and mechanism-oriented designs. Cross-lagged panel models, random-intercept cross-lagged models, growth mixture models, and intensive longitudinal designs can help separate between-person differences from within-person developmental changes. Experimental and quasi-experimental designs, such as parenting intervention trials, can also provide stronger evidence for causal inference (McAloon and Armstrong, 2024). In addition, studies should examine mediating mechanisms such as emotion regulation, psychological need frustration, self-blame, attachment insecurity, and social avoidance, while testing moderators such as age, gender, temperament, culture, and parental mental health. This would allow future research to move from identifying associations to explaining developmental pathways.

### 4.3 Challenge 3: translational barriers and the need for multimodal solutions

A third challenge concerns translation from research to real-world assessment and intervention. Parental psychological control is difficult to detect because it often appears in socially acceptable forms. Parents may present guilt induction as moral education, love withdrawal as discipline, and intrusive parenting as protection. Children may also appear well-adjusted because they have learned to comply with parental expectations. This creates a major translational barrier: families, schools, and clinicians may fail to recognize psychological control until children develop more visible emotional or behavioral problems (Costa et al., 2025; Song et al., 2025).

Traditional assessment tools may not be sensitive enough to detect this hidden pattern. A child may verbally report that family relationships are harmonious while showing subtle signs of distress during interaction, such as gaze avoidance, reduced vocal energy, delayed responses, facial tension, bodily withdrawal, or increased physiological arousal. Recent developmental and clinical research increasingly suggests that emotion regulation and mental health should be understood through multiple levels of analysis, including behavioral, emotional, physiological, and contextual indicators (Zitzmann et al., 2024; Konok et al., 2024). This is especially important for younger children, who may lack the verbal ability to describe psychological pressure, and for adolescents, who may strategically conceal distress to avoid conflict.

Multimodal assessment offers a promising solution. Future studies could combine parent–child interaction tasks with facial expression analysis, speech and language features, body movement, heart rate variability, electrodermal activity, and neural indicators of emotional reactivity and regulatory effort. Such methods may help identify the discrepancy between outward compliance and internal distress. For example, during a parent–child disagreement task, a child may agree with the parent verbally while displaying physiological arousal and reduced expressive variability. These patterns may provide more sensitive evidence of hidden emotional pressure than questionnaires alone (Gilgoff et al., 2024; Wentholt et al., 2025).

However, multimodal solutions must be implemented carefully. The use of video, audio, physiological signals, and AI-based analysis raises ethical concerns about privacy, consent, data security, interpretability, and potential misclassification. Therefore, future research should follow transparent and child-centered ethical standards. Multimodal models should not be used to label parents or children in a deterministic way; rather, they should support clinical judgment, family communication, and early intervention. Explainable models, preregistered protocols, culturally validated measures, and human-in-the-loop interpretation are essential for responsible use. Special consideration is also needed when collecting multimodal data from children and adolescents. Future studies should ensure appropriate parental consent and age-appropriate child assent, adopt robust data protection procedures, and carefully evaluate whether laboratory-based observations generalize to everyday family interactions. In addition, AI-derived emotional indicators should be interpreted cautiously, as multimodal signals may reflect multiple psychological processes and should not be treated as definitive evidence of a child's internal emotional state.

In terms of intervention, the most promising direction is not simply to reduce controlling behaviors, but to replace them with autonomy-supportive and emotionally validating parenting. Parents can be guided to set clear behavioral boundaries without manipulating children's emotions, to express disappointment without inducing shame, and to support decision-making without taking over children's agency (Bradshaw et al., 2025; McAloon and Armstrong, 2024). School-based and family-based programs may also help children develop emotional literacy, boundary awareness, assertive communication, and adaptive coping strategies. In this way, solutions should operate at both levels: reducing psychologically controlling parenting and strengthening children's socio-emotional resilience.

## 5 Conclusion

Parental psychological control represents a subtle but developmentally consequential form of parenting that affects children's socio-emotional development by intruding into their inner emotional and relational world. Unlike behavioral control, which may provide external structure and guidance, psychological control operates through emotional manipulation, conditional approval, relational threat, and excessive interference with children's autonomy. This review has argued that the hidden impact of parental psychological control cannot be fully understood by focusing only on outward behavioral adjustment or clinical symptoms. Instead, it is necessary to examine how psychologically controlling practices shape children's emotional autonomy, empathic expression, and social adaptation over time.

This review identified guilt induction, love withdrawal, and intrusive parenting as three representative forms of parental psychological control. Although these practices are often grouped under a single broad construct, they may influence children through distinct but interconnected developmental pathways. Guilt induction may weaken emotional autonomy by making children feel responsible for parental distress and by increasing self-blame, shame, and emotional over-responsibility. Love withdrawal may distort empathic expression by teaching children that affection is conditional upon obedience, thereby increasing relational anxiety and defensive sensitivity to others' emotions. Intrusive parenting may undermine social adaptation by limiting children's opportunities for independent decision-making, interpersonal problem solving, and age-appropriate exploration. Together, these mechanisms suggest that parental psychological control can produce a pattern of surface compliance but internal socio-emotional vulnerability.

A central argument of this review is that the effects of parental psychological control are often hidden. Children exposed to psychologically controlling parenting may appear obedient, mature, considerate, or well-adjusted, while internally experiencing suppressed emotions, weakened self-boundaries, fear of rejection, reduced agency, and interpersonal insecurity. This discrepancy between outward behavior and internal experience makes psychological control difficult to detect through traditional questionnaires or surface-level observation alone. Therefore, future research should move beyond global measures of parenting and adopt more fine-grained approaches that distinguish guilt induction, love withdrawal, intrusive interference, conditional regard, and related controlling practices.

The review also highlights several unresolved challenges in the field. Conceptually, parental psychological control needs to be more clearly differentiated from behavioral control, parental involvement, strictness, and autonomy support. Methodologically, the field remains overly dependent on self-report and cross-sectional designs, which limits the ability to identify hidden emotional processes and causal developmental pathways. Empirically, more longitudinal, observational, experimental, and culturally sensitive studies are needed to clarify how psychological control interacts with child age, temperament, gender, family stress, and sociocultural values. Addressing these challenges will help researchers move from documenting associations to explaining mechanisms.

A promising direction for future research is the integration of developmental psychology with multimodal affective and neurodevelopmental methods. Parent–child interactions are complex, dynamic, and emotionally layered. Children may verbally comply with parents while simultaneously showing subtle signs of distress through facial expression, vocal features, gaze behavior, body posture, physiological arousal, or neural indicators of regulatory effort. Multimodal assessment may therefore provide a more sensitive way to capture the implicit emotional burden of psychological control, especially among younger children or children who are reluctant to report distress directly. However, such methods should be implemented with careful attention to ethics, privacy, interpretability, and child-centered consent.

From a practical perspective, the findings reviewed here suggest that family education and intervention should not simply encourage parents to reduce harsh or controlling behaviors. More importantly, parents should be supported in developing autonomy-supportive and emotionally validating practices. This means setting clear behavioral boundaries without manipulating children's emotions, expressing concern without inducing guilt, maintaining warmth during disagreement, and allowing children to participate in age-appropriate decisions. Schools and mental health services can also help children strengthen emotional literacy, boundary awareness, assertive communication, and adaptive coping skills.
